# A230 THE ROLE OF THE MICROBIOTA IN NOCICEPTOR DEVELOPMENT AND PAIN SENSITIVITY

**DOI:** 10.1093/jcag/gwab049.229

**Published:** 2022-02-21

**Authors:** N Abdullah, M Defaye, A Hassan, M Cumenal, M Iftinca, D Young, C L Ohland, A Dufour, K McCoy, C Altier

**Affiliations:** Physiology and Pharmacology, University of Calgary Cumming School of Medicine, Calgary, AB, Canada

## Abstract

**Background:**

Pain is the most common cause of disability in IBD. What causes inter-individual variability in chronic pain after successful treatment of inflammation remains elusive. We have shown that activation of TRPV1+ colonic nociceptors is essential for the establishment of persistent pain in DSS colitis. Nociceptor development coincides with microbial colonization, while early life dysbiosis can lead to visceral hypersensitivity in adulthood. Whether the microbiota dictates nociceptor development and pain susceptibility remains unknown. Here we test the hypothesis that the microbiota programs nociceptor specification during early development, rendering them more susceptible to sensitization later in life. We have identified the aryl hydrocarbon receptor (AHR) that senses bacterial-derived metabolites as a candidate target that orchestrates transcriptional regulation in nociceptors.

**Aims:**

We investigated the developmental regulation of nociceptors by the microbiome and how it influences pain sensitivity. We will determine the effects of AHR activation on nociceptor lineage and function as well as the long term impact of AHR signaling on pain sensitivity.

**Methods:**

We have developed a germ-free (GF) TRPV1-GFP reporter mouse that was used to phenotype and visualise TRPV1+ nociceptors in the absence of a microbiota. We will isolate TRPV1+ neurons by FACS to identify genes that are under the control of the microbiota and to characterise the phosphoproteome of TRPV1+ nociceptors in GF conditions. Finally, we will investigate the role of AHR signaling in nociceptors both acutely and during development.

**Results:**

We showed a reduction in thermal pain threshold and a reduction in capsaicin test responses in GF mice. The number and size of DRG neurons was unchanged in GF mice. Examination of molecular markers for peptidergic (CGRP) and non-peptidergic (IB4) neurons did not show a difference. Finally, there was no difference in the expression of TRPV1, suggesting post-translational modification of the channel. In cultured DRG neurons, we found a decrease in capsaicin induced action potentials and a decrease in the amplitude of the capsaicin response in GF mice. Using RNAscope, we showed that TRPV1+ neurons express AHR.

**Conclusions:**

Our results highlight the importance of bacterial composition in regulating the development of nociceptors and pain sensitivity in adulthood. Furthermore, we are the first to demonstrate the expression of AHR in sensory neurons. These findings point to a role of the microbiota in programming nociceptors during development. My work will advance our understanding of the role of commensal bacteria in regulating pain and could lead to recommendations for the treatment of neonates in early life to reduce their risk of developing chronic pain later in life.

**Funding Agencies:**

CAG, CIHR

